# Significance of thermal relaxation on energy transport of Maxwell fluid rotating over a cylindrical surface with homogeneous–heterogeneous reactions

**DOI:** 10.1038/s41598-023-42679-w

**Published:** 2023-09-20

**Authors:** Jawad Ahmed, Faisal Nazir, Nevine M. Gunaime

**Affiliations:** 1 Department of Basic Sciences, University of Engineering and Technology, Taxila, 47050 Pakistan; 2 Department of Basic Sciences, College of Science and Theoretical Studies, Saudi Electronic University, Riyadh, 11673 Saudi Arabia

**Keywords:** Engineering, Materials science

## Abstract

Many industrial applications, including coating processes, roller bearing uses, and cooling gas turbine rotors, involve non-Newtonian fluid flow across rotating cylinders. The current study aims at evaluating the energy transport of the Maxwell fluid rotating over a horizontal cylindrical surface using the Cattaneo–Christov heat flux conduction model. This model predicts the properties of thermal relaxation by revising conventional Fourier's law. Isothermal cubic autocatalytic kinetics provides a homogeneous reaction, while first-order kinetics yields a heterogeneous reaction. With the help of transformations, the system of ODEs relating the equations for energy, momentum, and concentration is produced. For a numerical solution, the bvp4c scheme, which is based on the finite difference technique in Matlab 9.7 R2019b, is used. The importance of dominant parameters is displayed with the graphical depictions for axial, radial, and azimuthal flows, as well as temperature and concentration distributions. The noteworthy results illustrate that the Maxwell parameter has a declining influence on all velocity components. Further, thermal relaxation time causes a decline in the temperature field as well. Moreover, as the homogeneous–heterogeneous reaction parameters are increased, a reduction in fluid concentration is shown.

## Introduction

Manufacturing glass fibres, creating plastic films, growing crystals, cooling metal sheets, making paper, and many more engineering and industrial applications all depend on an understanding of non-Newtonian fluid movements. Rate type, integral type, and differential type are three categories used to describe the non-Newtonian model. The Maxwell fluid model^1^ is one of the most well-known rate type models due to its wide range of applications. For example, lubricants, polymer solutions, and crude oil are all considered to be examples of Maxwell fluid. Many scholars, including Shafique et al.^2^, focused on the Maxwell fluid motion in a rotating frame under the impact of chemical reactions and activation energy. Ali et al.^3^ studied the effects of buoyancy on the Falkner Skan flow of a Maxwell nanofluid past a wedge with binary activation energy and chemical reactions and obtained the numerical solution with FEM. Hayat et al.^4,5^ considered the stretched flow of Maxwell fluid under chemical reactions, MHD, and stagnation point region. Research on Maxwell fluid flow across various configurations and under different physical situations can be found in^6–13^.

Due to its wide range of technical mechanisms, including glass fibre, wire drawing, paper manufacture, and hot rolling, the flow created by rotating and stretching surfaces has drawn researchers to examine its features. The liquid movement over rotating cylinders is also crucial in a variety of applications, from axles and shafts to spinning projectiles. Numerical research on the axisymmetric movement of viscous fluid across a rotating and stretchable cylinder was conducted by Fang et al.^14^. Moreover, Fang et al.^15^ studied the constant flow over a revolving and stretching disk. Sprague and Weidman et al.^16^ investigated the flow of a viscous fluid caused by a purely rotating cylinder. Ahmed et al.^17^ explored the process of thermal transmission in the spinning Maxwell nanofluid produced by a revolving cylinder. Ahmed et al.^18^ came across the mixed convection phenomenon caused by stretching and vertically rotating a cylinder in a 3D Maxwell nanofluid flow. Titanium dioxide (TiO_2_) and aluminium oxide (Al_2_O_3_) as nanoparticles were used in Ahmed et al.'s^19^ analysis of the thermal characteristics of hybrid nanofluid motion across a swirling and stretchable cylinder. Due to the stretching and torsion of a cylinder, Ghoneim et al.^20^ concentrated on the nanofluid rotation with transmission of heat for SWCNT and MWCNT with the base liquid as water.

Differences in temperature between various systems generate the phenomenon known as heat transfer. The research on heat transfer mechanisms, such as convection, conduction, and radiation, is concerned with the exchange of momentum, mass, and energy. The application of this phenomenon is widespread in many different industries, including chemical reactions, polymer extrusion, and mechanical and nuclear engineering. Under certain conditions, Fourier's rule^21^ was examined as a very effective model for heat transmission mechanisms^22–27^. By including thermal relaxation time, Cattaneo^28^ updated Fourier's work. The result of this modification was the hyperbolic shape's energy equation. In the Maxwell–Cattaneo relationship, Christov^29^ extended Cattaneo's findings even further by using the upper-convective Oldroyd derivative, preferably the partial time derivative. Sui et al.^30^ considered the viscous flow of fluid with heat transmission using numerical modelling in a wave microchannel with a rectangular cross-section. Sheikholeslami et al.^31^ scrutinized the influence of thermal radiation on the movement of MHD nanofluid between the gap of two horizontally moving plates. According to the Cattaneo–Christov theory, Khan et al.^32^ examined the mass and heat diffusion for spinning Oldroyd-B fluid over the stretched sheet. The heat and flow transport inside a microchannel with a triangular part were numerically modelled by Rezaei et al.^33^. Irfan et al.^34,35^ took into consideration a bidirectional surface of stretching. With the use of the Cattaneo–Christov model, Han et al.^36^ investigated the flow of viscoelastic fluid. For the temperature and concentration fields, they discovered the graphical results. Using the Cattaneo–Christov theory and a stretched sheet, Upadhya et al.^37^ looked into the MHD flow of a viscous fluid. With higher levels of the thermal relaxation number, there is a reduction in heat transmission. Farooq et al.^38^ explored the Cattaneo–Christov theory when addressing viscous fluid flow with variable mass diffusivity and thermal conductivity. According to their findings, concentration and temperature distribution decrease as mass and thermal relaxation time parameters increase. Also, multiple studies have been done to analyse the Cattaneo–Christov theory for heat transportation in the flow of different fluid models (see Refs.^39–43^).

Chemical processes are classified as homogeneous or heterogeneous depending on whether they occur in a large volume of fluid (homogeneous) or on specific catalytic surfaces (heterogeneous). Many chemical reaction systems, such as catalysis, biological processes, and combustion, include both homogeneous–heterogeneous reactions. It is typical for the connection between chemical reactions to be quite complex since they include the consumption and synthesis of reactant species at various rates both on the catalytic surfaces and inside the fluid. Khan et al.^44^ considered the fluid model of Maxwell across a spinning disk and examined the influence of homogeneous–heterogeneous processes, convective boundary conditions, and nonlinear thermal radiations. Hayat et al.^45^ observed the effects of homogeneous–heterogeneous reactions and thermal stratification on the Jaffrey fluid rotating in the region occupied between doubly rotating disks. By considering the Maxwell model, the efficiency of homogeneous–heterogeneous reactions between the two rotating parallel disks with enhanced heat conduction theory was inspected by Ahmed et al.^46^. In the presence of homogeneous–heterogeneous reactions, the time dependent and magnetized motion of Maxwell liquid on a rotating and a vertically moving disk were studied by Khan et al.^47^.

The available literature indicates that no research has been done on the features of mass and heat transfer with homogeneous–heterogeneous reactions for 3D Maxwell fluid motion using Cattaneo–Christov theory. This study incorporates each aspect that is lacking in previously published work. The paper further elaborates on the significance of Lorentz forces and convective boundary conditions on thermal and solutal transports. With the use of momentum, temperature, and concentration conservation equations, the governing 3D flow physical problem is modelled and numerically solved with the bvp4c solver in Matlab. By illustrating and elaborating on graphical and tabular trends, the effect of the active parameters is strongly highlighted. This article is organized as: the flow arrangement and mathematical model of the rotating cylinder are presented in “Problem Formulation”. Section “Numerical solution” presents the method to the solution. Graphical illustrations and a table in “Discussion” show the physical significance of the relevant factors on flow, thermal, and mass transport. Section “Conclusions” concludes by summarising the outcomes obtained.

## Problem formulation

**Figure 1:**
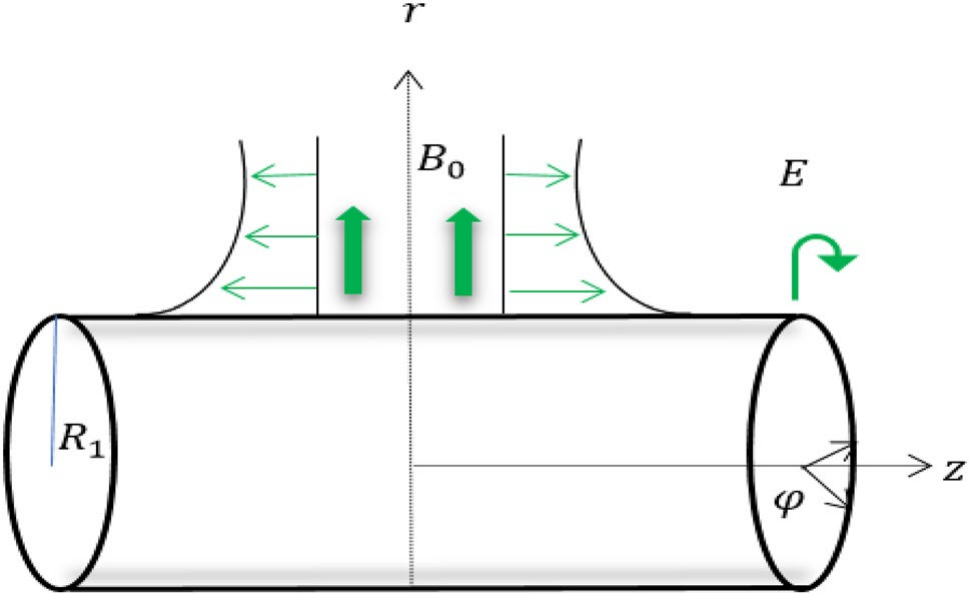
Flow configuration.

Suppose a rotating cylinder with a radius of ${R}_{1}$ that induces a Maxwell fluid rotating flow which is electrically conducting in the existence of a transverse magnetic field. Assuming that the cylinder rotates around its axis at a constant speed and that the axial distance has a linear relationship with the cylinder's stretching velocity, as shown in Fig. 1. The cylindrical coordinated system $(z,\varphi ,r)$ is used to represent the mathematical explanation of the physical problem. On the assumption that the induced electric and magnetic fields are to be ignored, a uniform magnetic beam with strength ${B}_{0}$ is applied along the *r*-direction. By assuming that $T(z, {R}_{1}) = {T}_{w}$ for the temperature at the cylinder's surface, thermal analysis is examined. The Cattaneo–Christov heat flow theory and convective condition are applied when the analysis of heat transport is taken into account.

In addition, the homogeneous and heterogeneous reactions are considered. The cubic autocatalytic homogeneous reaction is

1 $$A+2B\to 3B,\quad \text{r}={k}_{c}a{b}^{2},$$

The first-order isothermal reaction can be represented in the following form:

2 $$A\to B, \quad \text{r} ={k}_{s}a,$$

here (${k}_{c}, {k}_{s}$) are the rate constants, $(A, B)$ represent the chemical species, and $(a, b)$ represent the concentrations of those species. Moreover, we presume that this chemical reaction is isothermal. The leading equation of the Maxwell 3D flow in the occurrence of the magnetic field is:

3 $$\nabla \cdot {\varvec{V}}\boldsymbol{ }= 0,$$

4 $$\rho \left({\varvec{V}}.\nabla \right){\varvec{V}}=-\nabla p+\nabla \cdot {\varvec{S}}+{{\varvec{J}}}_{1}\times {\varvec{B}}$$

5 $$\rho {c}_{p}\left({\varvec{V}}.\nabla \right)T=-\nabla \cdot {\varvec{q}},$$

6 $$\left({\varvec{V}}.\nabla \right)a={D}_{A}{\nabla }^{2}a-{k}_{c}a{b}^{2},$$

7 $$\left({\varvec{V}}.\nabla \right)b={D}_{B}{\nabla }^{2}b+{k}_{c}a{b}^{2},$$

In the case of a Maxwell fluid, the extra stress tensor ***S*** is defined as:

8 $$\left(1 + {\lambda }_{1}\frac{D}{Dt}\right){\varvec{S}} = \mu {{\varvec{A}}}_{1},$$

where ${\lambda }_{1}$ is the time of relaxation, *µ* dynamic viscosity, $T$ liquid temperature, ${c}_{p}$ heat capacity at constant pressure, ${{\varvec{J}}}_{1}$ current density, and ${D}_{A}$ and ${D}_{B}$ are the diffusion coefficients. Further ${{\varvec{A}}}_{1}$ indicates the first tensor of Rivlin–Ericksen and $\frac{D}{Dt}$ the upper convective derivative written as

9 $${{\varvec{A}}}_{1} = \nabla {\varvec{V}} + {\left(\nabla {\varvec{V}} \right)}^{t},$$

10 $$\frac{D{\varvec{S}}}{Dt}=\frac{\partial {\varvec{S}}}{\partial t}+\left({\varvec{V}}.\nabla \right){\varvec{S}}-{\varvec{L}}{\varvec{S}}-{\varvec{S}}{{\varvec{L}}}^{t}.$$

According to the steady, incompressible and axisymmetric suppositions, the governing equations for the current energy and flow transfer problem are given below

11 $$\frac{\partial u}{\partial r}+\frac{\partial w}{\partial z}+\frac{u}{r}=0,$$

12 $$u\frac{\partial u}{\partial z}+w\frac{\partial u}{\partial r}+{\lambda }_{1}\left({u}^{2}\frac{{\partial }^{2}u}{\partial {z}^{2}}+2uw\frac{{\partial }^{2}u}{\partial r\partial z}+{w}^{2}\frac{{\partial }^{2}u}{\partial {r}^{2}}\right)=v\left(\frac{{\partial }^{2}u}{\partial {r}^{2}}+\frac{1}{r}\frac{\partial u}{\partial r}\right)-\frac{\sigma {B_{0}}^{2}}{\rho }\left(u+{\lambda }_{1}w\frac{\partial u}{\partial r}\right),$$

13 $$\begin{aligned} & u\frac{\partial v}{\partial z}+w\frac{\partial v}{\partial r}+\frac{wv}{r}{+\lambda }_{1}\left({u}^{2}\frac{{\partial }^{2}v}{\partial {z}^{2}}+2uw\frac{{\partial }^{2}v}{\partial r\partial z}+{w}^{2}\frac{{\partial }^{2}v}{\partial {r}^{2}}+\frac{2wv}{r}\frac{\partial w}{\partial r}+\frac{2uv}{r}\frac{\partial w}{\partial z}-\frac{2{w}^{2}v}{{r}^{2}} \right)\\ &\quad=v\left(\frac{{\partial }^{2}v}{\partial {r}^{2}}-\frac{v}{{r}^{2}}+\frac{1}{r}\frac{\partial v}{\partial r}\right)-\frac{\sigma {B_{0}}^{2}}{\rho }\left(v+{\lambda }_{1}w\frac{\partial v}{\partial r}-{\lambda }_{1}\frac{wv}{r}\right),\end{aligned}$$

14 $$\rho {c}_{p}\left(u\frac{\partial T}{\partial z}+w\frac{\partial T}{\partial r}\right)=-\nabla .{\varvec{q}},$$

15 $$u\frac{\partial a}{\partial z}+w\frac{\partial a}{\partial r}={D}_{A}\left(\frac{{\partial }^{2}a}{\partial {r}^{2}}+\frac{1}{r}\frac{\partial a}{\partial r}\right)-{k}_{c}a{b}^{2},$$

16 $$u\frac{\partial b}{\partial z}+w\frac{\partial b}{\partial r}={D}_{B}\left(\frac{{\partial }^{2}b}{\partial {r}^{2}}+\frac{1}{r}\frac{\partial b}{\partial r}\right)+{k}_{c}a{b}^{2},$$

with the boundary conditions (BCs)

17 $$u=2az, v=E, w=0, -k\frac{\partial T}{\partial r}={h}_{t}\left({T}_{f}-T\right), {D}_{{\varvec{A}}}\frac{\partial a}{\partial r}={k}_{s}a, {D}_{{\varvec{B}}}\frac{\partial b}{\partial r}={-k}_{s}a\quad \mathrm{at }\quad r={R}_{1},$$

18 $$u=0, v=0,T\to {T}_{\infty }, a\to {a}_{0},b\to 0\quad \mathrm{ at }\quad r\to \infty .$$

In Eq. (14), ${\varvec{q}}$ is the heat flux satisfying the relation

19 $${\varvec{q}}+{\delta }_{E}\left(\frac{\partial {\varvec{q}}}{\partial t}+{\varvec{V}}\cdot \nabla {\varvec{q}}-{\varvec{q}}\cdot \nabla {\varvec{V}}+\left(\nabla \cdot {\varvec{V}}\right){\varvec{q}}\right)=-k\nabla T,$$

where thermal conductivity is represented by $k$, and thermal relaxation time by ${\delta }_{E}$. We get the following expression when ${\varvec{q}}$ is eliminated from Eqs. (14) and (19):

20 $$\begin{aligned}\left(u\frac{\partial T}{\partial z}+w\frac{\partial T}{\partial r}\right)&=\alpha\left(\frac{{\partial }^{2}T}{\partial {r}^{2}}+\frac{1}{r}\frac{\partial T}{\partial r}\right)-{\delta }_{E}\left[{u}^{2}\frac{{ \partial }^{2}T}{\partial {z}^{2}}+{w}^{2}\frac{{\partial }^{2}T}{\partial {r}^{2}}+2uw\frac{{\partial }^{2}T}{\partial r\partial z}\right. \\ & \quad \left.+\frac{\partial T}{\partial z}\left(u\frac{\partial u}{\partial z}+w\frac{\partial u}{\partial r}\right)+\frac{\partial T}{\partial r}\left(u\frac{\partial w}{\partial z}+w\frac{\partial w}{\partial r}\right)\vphantom{{u}^{2}\frac{{ \partial }^{2}T}{\partial {z}^{2}}}\right].\end{aligned}$$

The following transformation group is introduced (Ref.^14^):

21 $$\eta =\frac{{r}^{2}}{{R}_{1}^{2}}, u=2az{f}^{\prime}\left(\eta \right),v=Eg\left(\eta \right), w=-a{R}_{1}\frac{f\left(\eta \right)}{{\eta }^\frac{1}{2}},$$

22 $$\theta \left(\eta \right)=\frac{T-{T}_{\infty }}{{T}_{f}-{T}_{\infty }},\phi \left(\eta \right)=\frac{a}{{a}_{0}}, \psi \left(\eta \right)=\frac{b}{{a}_{0}}.$$

When the preceding ansatz is used, Eq. (11) automatically becomes satisfied, and Eqs. (12, 13, 15–18, 20) yield.

23 $$\eta {f}^{{\prime}{\prime}{\prime}}+{f}^{{\prime}{\prime}}+Ref{f}^{{\prime}{\prime}}-{Ref}^{{\prime}2}-\beta Re\left(\frac{{f}^{2}{f}^{{\prime}{\prime}}}{\eta }+2{f}^{2}{f}^{{\prime}{\prime}{\prime}}-4f{f}^{\prime}{f}^{{\prime}{\prime}}\right)-MRe\left(\frac{{f}^{\prime}}{2}-\beta f{f}^{{\prime}{\prime}}\right)=0,$$

24 $$2{\eta }^{2}{g}^{{\prime}{\prime}}+2\eta {g}^{\prime}-\frac{g}{2}+2Re\eta f{g}^{\prime}+Refg-\beta Re\left(2{f}^{2}{g}^{\prime}+4\eta {f}^{2}{g}^{{\prime}{\prime}}+4f{f}^{\prime}g-\frac{4{f}^{2}g}{\eta }\right)-MRe\left(\eta g-2\beta \eta f{g}^{\prime}-\beta fg\right)=0,$$

25 $${\eta \theta }^{{\prime}{\prime}}+{\theta }^{\prime}+RePrf{\theta }^{\prime}-{\beta }_{t}RePr({f}^{2}{\theta }^{{\prime}{\prime}}+f{f}^{\prime}{\theta }^{\prime} )=0,$$

26 $$\frac{1}{{S}_{c}}\left(\eta {\phi }^{{\prime}{\prime}}+{\phi }^{\prime}\right)+Re(f{\phi }^{\prime}-{k}_{1}\phi {\psi }^{2})=0,$$

27 $$\frac{\delta }{{S}_{c}}\left(\eta {\psi }^{{\prime}{\prime}}+{\psi }^{\prime}\right)+Re(f{\psi }^{\prime}+{k}_{1}\phi {\psi }^{2})=0,$$

with BCs.

28 $$\begin{aligned} & f\left(1\right)=0, {f}^{\mathrm{^{\prime}}}\left(1\right)=1, g\left(1\right)=1, {\theta }^{{^{\prime}}}\left(1\right)=-\gamma \left(1-\theta \left(1\right)\right), {\phi }^{{^{\prime}}}\left(1\right)={k}_{2}\phi \left(1\right), {\delta \psi }^{{^{\prime}}}\left(1\right)={-k}_{2}\phi \left(1\right), \\ &\quad {f}^{\prime}\left(\infty \right)=0, g\left(\infty \right)=0, \theta \left(\infty \right)=0, \phi \left(\infty \right)=1, \psi \left(\infty \right)=0 \, ,\end{aligned}$$

where $\beta ={\lambda }_{1}a$ is the Maxwell number, $M=\frac{\sigma {B_{0}}^{2}}{\mathrm{a}\rho }$ the magnetic variable, $Re=\frac{a{R}_{1}^{2}}{2v}$ the Reynolds parameter, $Pr=\frac{v}{\alpha }$ the Prandtl variable, $\gamma =\frac{h_t}{k}\sqrt{\frac{v}{a}}$ the Biot number, ${\beta }_{t}= 2a{\delta }_{E}$ the thermal relaxation time parameter, ${k}_{1}=\frac{{k}_{c}{a}_{0}^{2}}{2a}$ homogeneous reaction, ${S}_{c}=\frac{v}{{D}_{A}}$ the Schmidt parameter, ${k}_{2}=\frac{{k}_{s}{R}_{1}}{2{D}_{A}}$ the heterogeneous reaction parameter and $\delta =\frac{{D}_{B}}{{D}_{A}}$ is the diffusion coefficients ratio.

Assuming that the diffusion coefficients (${D}_{A}, {D}_{B}$) are equal in size. This hypothesis leads to an examination of chemical reactions when ${D}_{A}$ and ${D}_{B}$ are equivalent, i.e. $\delta =1$. Based on this assumption, the following correlation can be derived:

29 $$\phi \left(\eta \right)+ \psi \left(\eta \right)=1.$$

Thus, the Eqs. (26) and (27) turn into

30 $$\frac{1}{{S}_{c}}{(\eta \phi }^{{\prime}{\prime}}+{\phi }^{\prime}) +Ref{\phi }^{\prime} -Re\; {k}_{1}\phi {\left(1-\phi \right)}^{2}=0,$$

with BCs

31 $${\phi }^{\mathrm{^{\prime}}}\left(1\right)={k}_{2}\phi (1), \phi \left(\infty \right)=1.$$

To achieve the fast convergence, the variable $\eta$ is converted as $\eta ={e}^{x}$ as followed by Fang and Yao^14^. Hence, Eqs. (23)–(25), (28), (30) and (31) become

32 $${f}_{xxx}-2{f}_{xx}+{f}_{x}-Re\left({f}_{x}^{2}-f{f}_{xx}+f{f}_{x}\right)-\beta Re{e}^{-x}\left(2{f}^{2}{f}_{xxx}-5{f}^{2}{f}_{xx}+3{f}^{2}{f}_{x}-4f{f}_{x}{f}_{xx}+4f{f}_{x}^{2}\right)-MRe\left({e}^{x}\frac{{f}_{x}}{2}-\beta f{f}_{xx}+\beta f{f}_{x}\right)=0,$$

33 $$2{g}_{xx}-\frac{g}{2}+Re\left(2f{g}_{x}+fg\right)-\beta Re{e}^{-x}\left(2{f}^{2}{g}_{x}+4{f}^{2}{g}_{xx}+4{f}^{2}{g}_{x}+4f{f}_{x}g-4{f}^{2}g\right) -MRe\left({e}^{x}g-2\beta f{g}_{x}-\beta gf\right)=0,$$

34 $$\left(1-{\beta }_{t}RePr{e}^{-x}{f}^{2}\right){\theta }_{xx}+RePrf{\theta }_{x}-{\beta }_{t}RePr(f{f}_{x}{\theta }_{x}-{e}^{-x}{f}^{2}{\theta }_{x})=0,$$

35 $${\frac{1}{{S}_{c}}\phi }_{xx}+Re\left(f{\phi }_{x}-{e}^{x}{k}_{1}\phi {(1-\phi )}^{2} \right)=0,$$

with the transformed BCs

$$f\left(0\right)=0, {f}_{x}\left(0\right)=1, g\left(0\right)=1, {\theta }_{x}\left(0\right)=-\gamma \left(1-\theta \left(0\right)\right), {\phi }_{x}\left(0\right)={k}_{2}\phi \left(0\right),$$

36 $${f}^{\prime}\left(\infty \right)=0, g\left(\infty \right)=0, \theta \left(\infty \right)=0, \phi \left(\infty \right)=1.$$

The dimensionless form of Nusselt number in the limiting case when ${\delta }_{E}=0$ is defined as

37 $$Nu=-2{\theta }^{\prime}\left(1\right).$$

## Numerical solution

**Figure 2:**
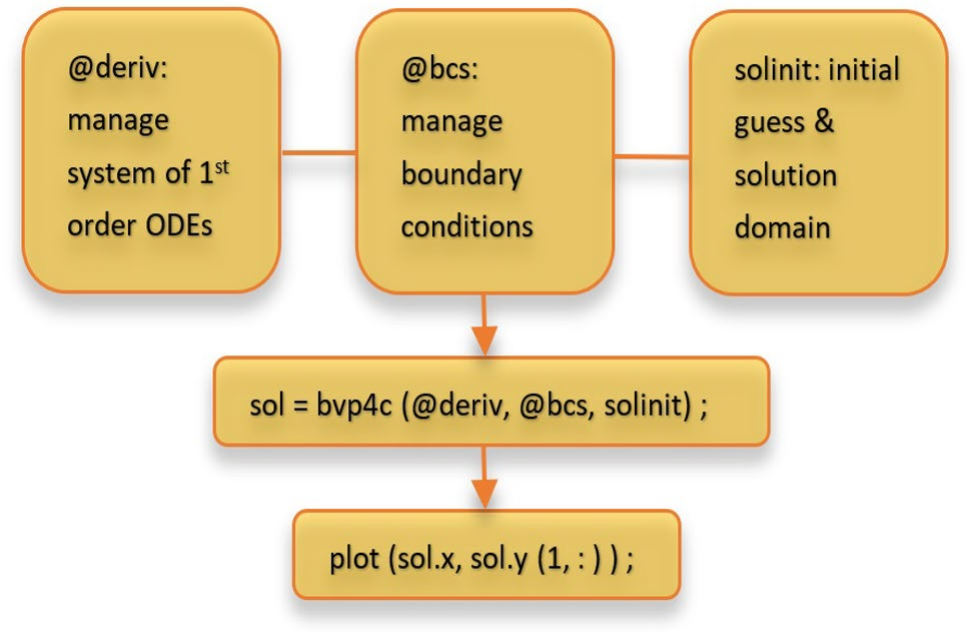
Flow diagram of bvp4c technique.

Using the bvp4c solver in Matlab 9.7 R2019b, the coupled system of Eqs. (32–35) and (36) demonstrating energy, momentum, temperature, and concentration with conditions is numerically integrated. The use of Lobatto IIIA formula^48,49^ is the central idea of the bvp4c solver. It is crucial to have initial iterations that fulfil the boundary conditions to approximate the solution. Once these initial guesses have been established, the iteration process proceeds by modifying the initial guess using an alternative technique known as the finite difference method. The working mechanism of this scheme is illustrated in the flow diagram as shown in Fig. 2, which provides a visual representation of the steps involved in the numerical integration process for solving the equations.

We define the new variables below with this in mind. Let, $f={R}_{1}$, ${f}_{x}={R}_{2}$, ${f}_{xx}={R}_{3}$, ${f}_{xxx}=R{R}_{1}$, $g={R}_{4}$, ${g}_{x}={R}_{5}$, ${g}_{xx}=R{R}_{2}$, $\theta ={R}_{6}$, ${\theta }_{x}={R}_{7}$, ${\theta }_{xx}=R{R}_{3}, \phi ={R}_{8}$, and ${\phi }_{x}={R}_{9}$, ${\phi }_{xx}=R{R}_{4}$ for Eqs. (32)–(36). The first-order ODEs that result are listed below:

38 $$R{R}_{1}=\left(2{R}_{3}-{R}_{2}+Re\left({R}_{2}^{2}-{R}_{1}{R}_{3}+{R}_{1}{R}_{2}\right)+\beta Re{e}^{-x}\left(3{R}_{1}^{2}{R}_{2}-5{R}_{1}^{2}{R}_{3}-4{R}_{1}{R}_{2}{R}_{3}+4{R}_{1}{R}_{2}^{2}\right)+MRe\left({e}^{x}\frac{{R}_{2}}{2}-\beta {R}_{1}{R}_{3}+\beta {R}_{1}{R}_{2}\right)\right)/{{C}_{1}},$$

39 $$R{R}_{2}=\left(\frac{{R}_{4}}{2}-Re\left(2{R}_{1}{R}_{5}+{R}_{1}{R}_{4}\right)+\beta Re{e}^{-x}\left(6{R}_{1}^{2}{R}_{5}+4{R}_{1}{R}_{2}{R}_{4}-4{R}_{1}^{2}{R}_{4}\right)+MRe\left({e}^{x}{R}_{4}-2\beta {R}_{1}{R}_{5}-\beta {R}_{1}{R}_{4}\right)\right)/{{C}_{2}},$$

40 $$R{R}_{3}=\left({\beta }_{t}RePr\left({R}_{1}{R}_{2}{R}_{7}-{e}^{-x}{R}_{1}^{2}{R}_{7}\right)-RePr{R}_{1}{R}_{7}\right)/{{C}_{3}},$$

41 $$R{R}_{4}=-Re{S}_{c}{R}_{1}{R}_{9}+{Re {S}_{c}e}^{x}{k}_{1}{R}_{8}{\left(1-{R}_{8}\right)}^{2}),$$

where

$${C}_{1}=1-2\beta Re{e}^{-x}{R}_{1}^{2}, {C}_{2}=2-4\beta Re{e}^{-x}{R}_{1}^{2}, {C}_{3}=\left(1-{\beta }_{t}RePr{e}^{-x}{R}_{1}^{2}\right),$$

with BCs

42 $${R}_{1}\left(0\right)=0,{R}_{2}\left(0\right)=1,{R}_{4}\left(0\right)=1,{R}_{7}\left(0\right)=-\gamma \left(1-{R}_{6}\left(0\right)\right), {R}_{9}\left(0\right)={k}_{2}{R}_{8}\left(0\right),{R}_{2}\left(\infty \right)=0, {R}_{4}\left(\infty \right)=0, {R}_{6}\left(\infty \right)=0, {R}_{8}\left(\infty \right)=1.$$

## Discussion

This section reveals the numerical results with a physical explanation for flow, thermal, and solute transports under the influence of the relevant physical parameters, such as the Biot number $\gamma$, Prandtl number $Pr$, Maxwell parameter $\beta$, magnetic parameter $M$, chemical reaction parameters (${k}_{1}$, ${k}_{2}$), and Schmidt number ${S}_{c}$. We fix the values of relevant quantities for graphical representations during the numerical computation as $Re=2.0, M=0.5, \beta =0.01$, $Pr=2.0$, ${\beta }_{t}=0.2$, ${S}_{c}=0.4$, ${k}_{1}=0.1$, ${k}_{2}=0.2$, $\gamma =0.5$.

**Figure 3:**
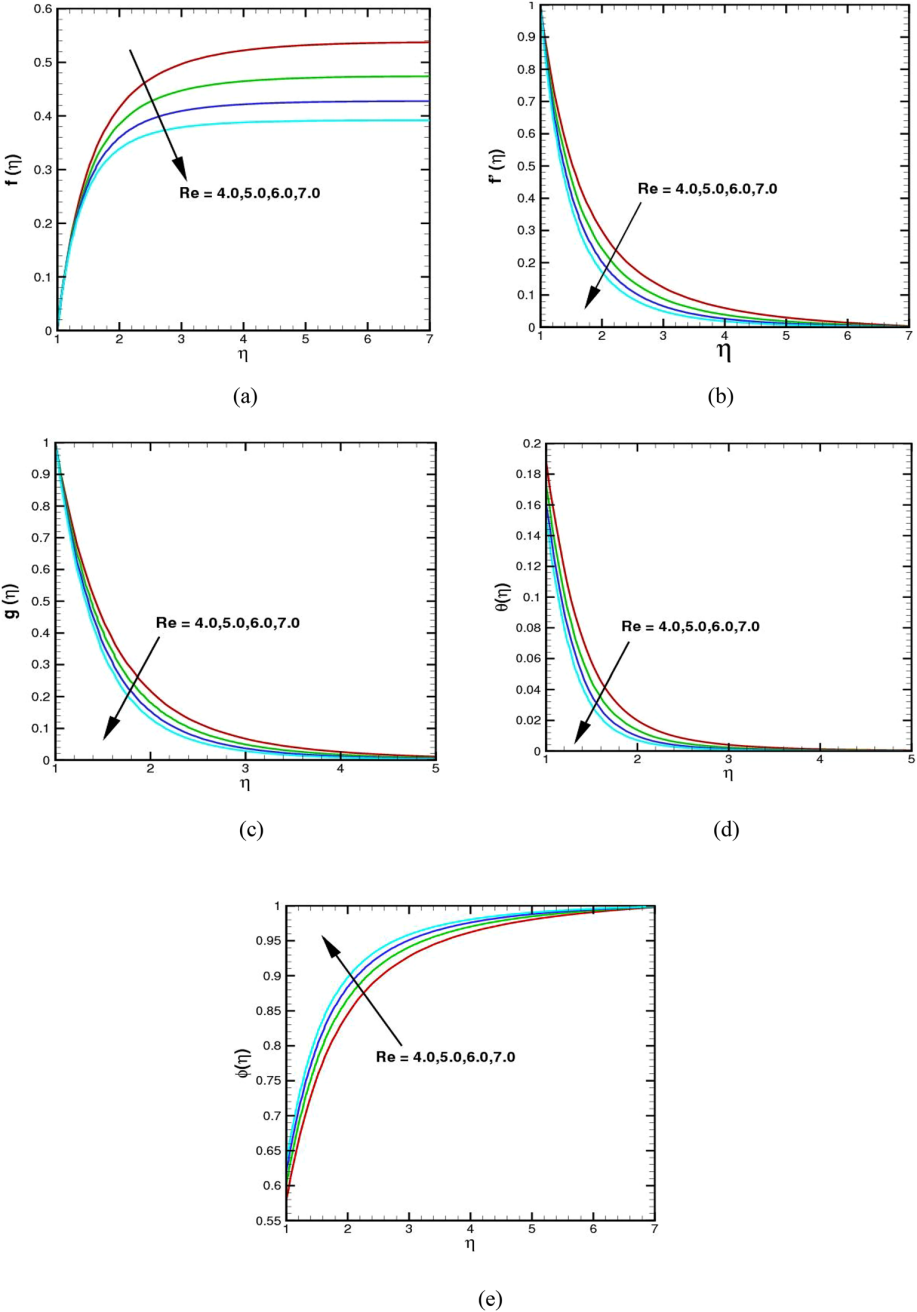
(**a**–**e**) The $f(\eta )$,${f}^{\prime}(\eta )$, $g(\eta )$, $\theta (\eta )$, and $\phi (\eta )$ via $Re.$

The impact of the Reynolds number $Re$ on the radial velocity $f(\eta )$, axial velocity ${f}^{\prime}(\eta )$, aximuthul velocity $g(\eta )$, temperature field $\theta (\eta )$, and concentration distribution $\phi (\eta )$ is shown in Fig. 3a–e. It demonstrates that the flow fields in Fig. 3a–c degrade and flow only occurs close to the surface at increasing values of $Re$. The Reynolds number (Re) is a dimensionless value utilized to identify the characteristics of fluid motion. When the Reynolds number is between 4 and 7, the flow is typically considered to be transitional. The values of the profiles for axial, radial, tangential, and temperature decline as the $Re$ upsurges, while the concentration of the fluid rises. Physically, greater values of $Re$ cause a rise in the inertial force in the fluid flow. The inertial force acts as an opposing force to the fluid flow agent, causing the flow field to contract in every direction. We are aware that as $Re$ increases, the forced convection mechanism in the flow decreases, resulting in a decrease in the temperature field.

**Figure 4:**
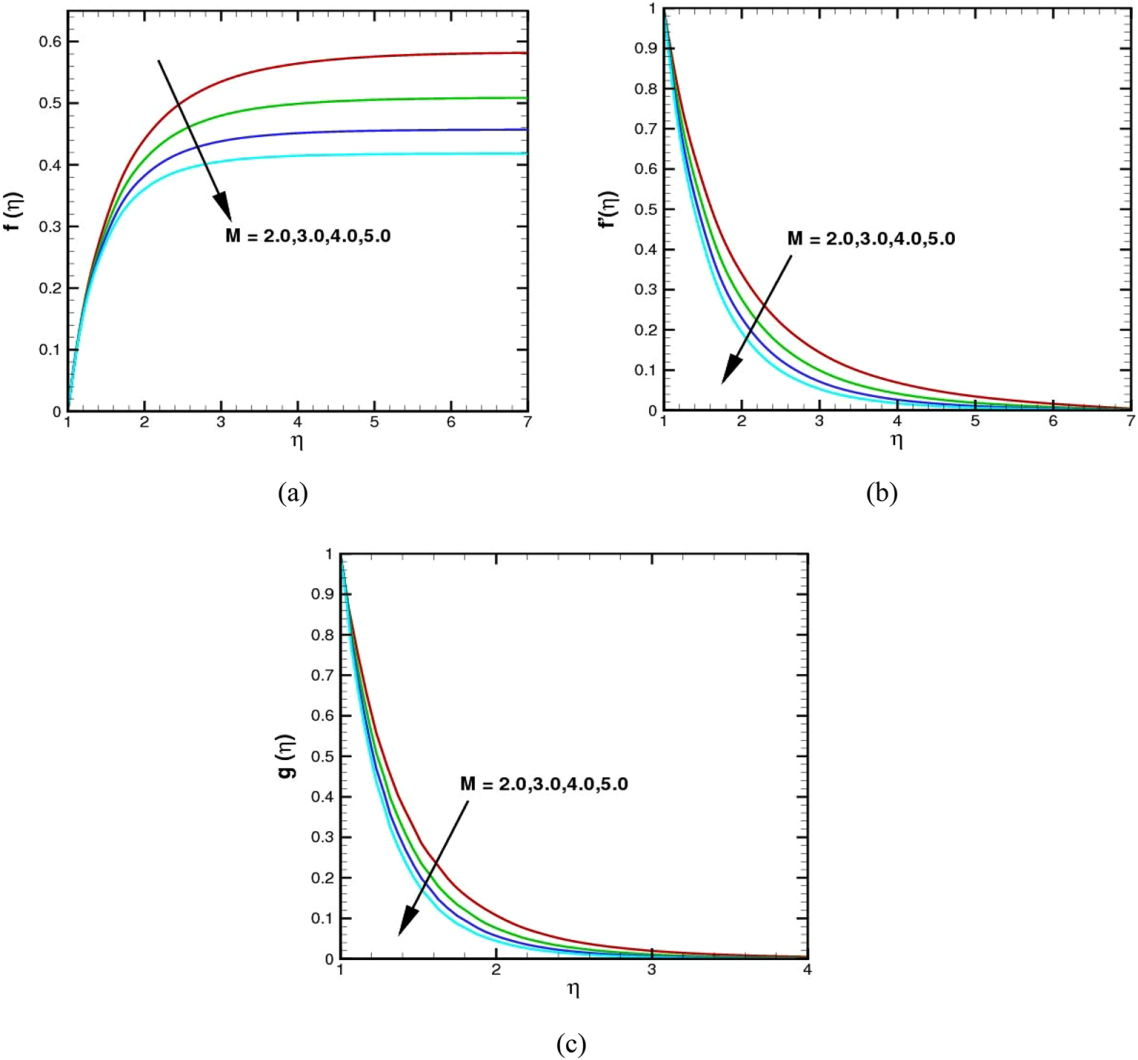
(**a**–**c**) The $f(\eta )$, ${f}^{\prime}(\eta )$, and $g(\eta )$ via $M$.

The influence of the $M$ on the radial, axial, and azimuthal velocities is reported in Fig. 4a–c. It demonstrates that an upsurge in the magnetic parameter $M$ causes a fall in fluid motion. The magnetic number $M$ is a measure of the ratio of the magnetic force to the inertial force on a particle in a rotating fluid. Theses graphs show a decrease in velocities as the magnetic number increases from 2 to 5. This is because as the magnetic forces increase, they tend to slow down or stop the particles in the rotating fluid.

**Figure 5:**
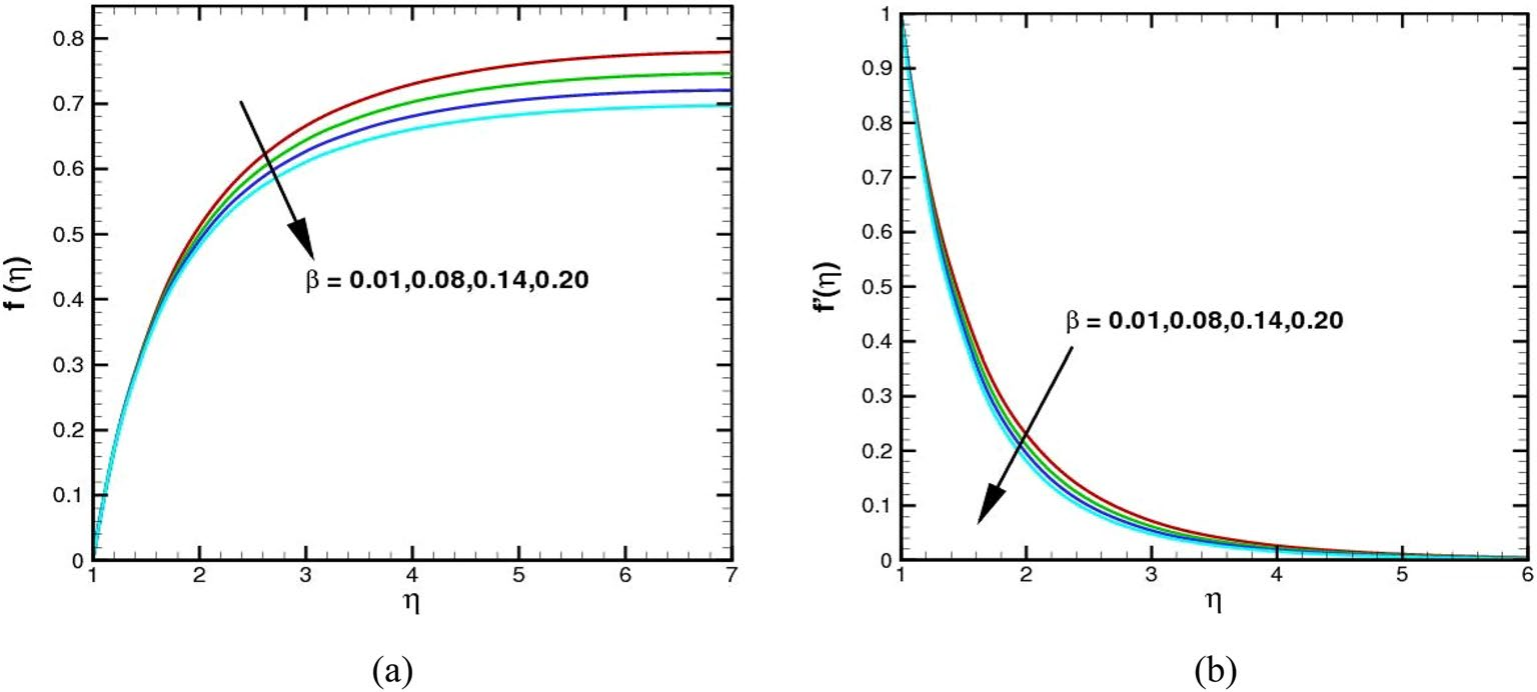
(**a**,**b**) The $f\left(\eta \right)$ and ${f}^{\prime}(\eta )$ via $\beta$.

The effect of the Maxwell number $\beta$ on the radial velocity $f(\eta )$ and axial velocity ${f}^{\prime}(\eta )$ on the flow field is shown in Fig. 5a–b. The value of $\beta$ is an indicator of the strength of a fluid's circulation. As it increases, the axial velocity of the fluid decreases as well, while the radial velocity of the fluid also drops. This happens because the axial velocity is affected by the circulation of the fluid, while the radial velocity is affected by the centrifugal force of the fluid. Physically, the rheology of a material of the viscoelastic type is defined by $\beta$. The dimensionless relaxation time is the Maxwell parameter $\beta$. These phenomena are depicted using relaxation time. Therefore, as the Maxwell number upsurges, both the radial and the axial velocity decline.

**Figure 6:**
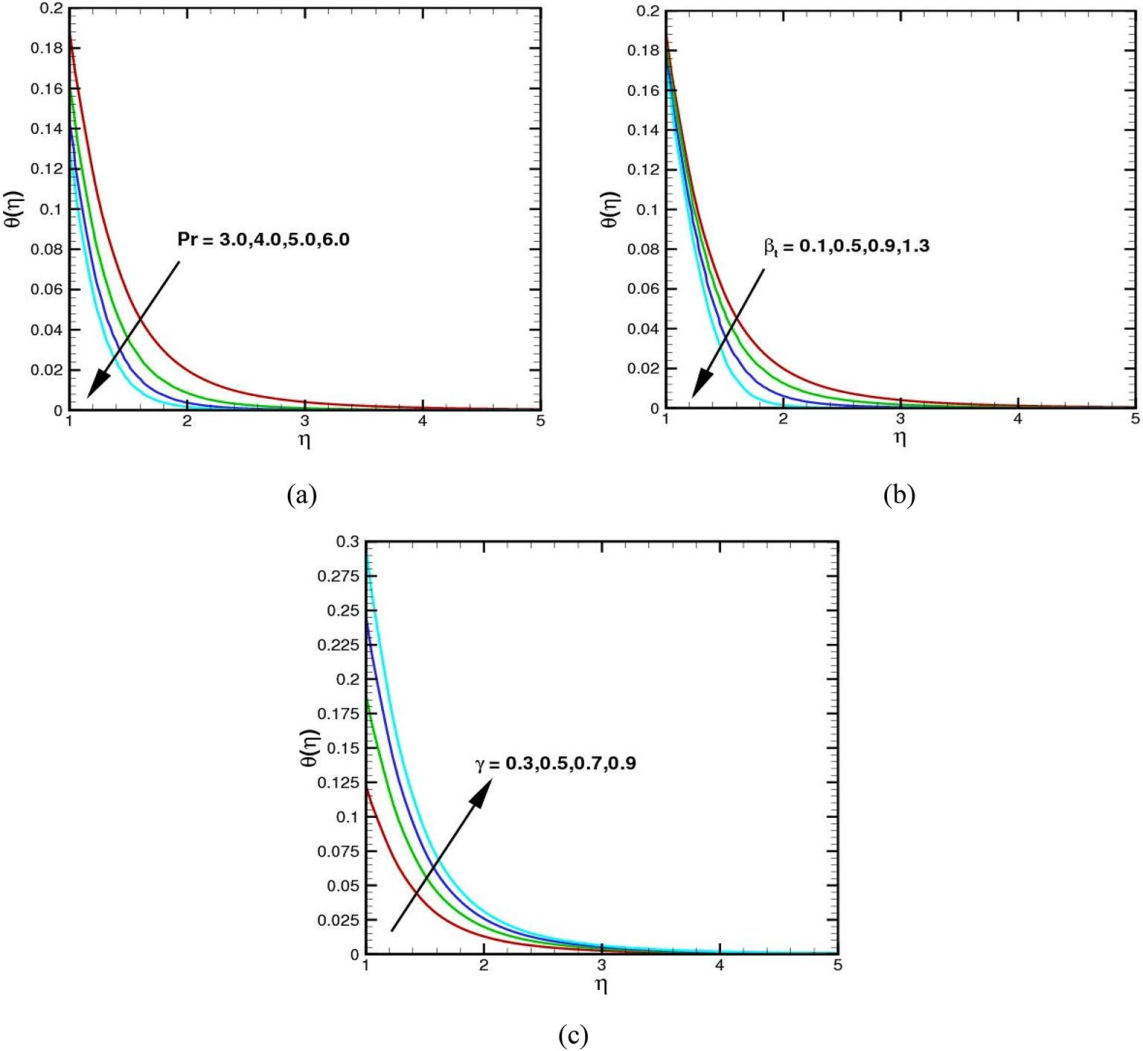
(**a**–**c**) The $\theta (\eta )$ via $Pr$, ${\beta }_{t}$ and $\gamma$.

Figure 6a–c show the impact of the Prandtl variable $Pr$, thermal relaxation time ${\beta }_{t}$, and Biot number $\gamma$ on the temperature profile. The Prandtl number, a dimensionless number, is a measure of a fluid's momentum diffusivity to thermal diffusivity ratio. A graph of the temperature profile in Fig. 6a versus the Prandtl number will show how the temperature increases or decreases as the $Pr$ rises. As the number Pr increases, the $\theta (\eta )$ decreases. This graph also indicates that the temperature will decrease faster in comparison to the rate at which the $Pr$ upsurges. Thermal relaxation time is the amount of time it takes for a material to reach a given fraction of its equilibrium temperature after a temperature change. The decrease in the graph of the temperature profile in Fig. 6b indicates that the material is slowly cooling off as time passes. This is due to the thermal relaxation time; the higher the value, the longer it will take for the temperature to decrease. The increase in the graph of temperature profile in Fig. 6c is a representation of the relationship between the rate of heat transfer and the Biot number in a system. As the $\gamma$ rises, the resistance to heat transfer upsurges and the temperature profile increases.

**Figure 7:**
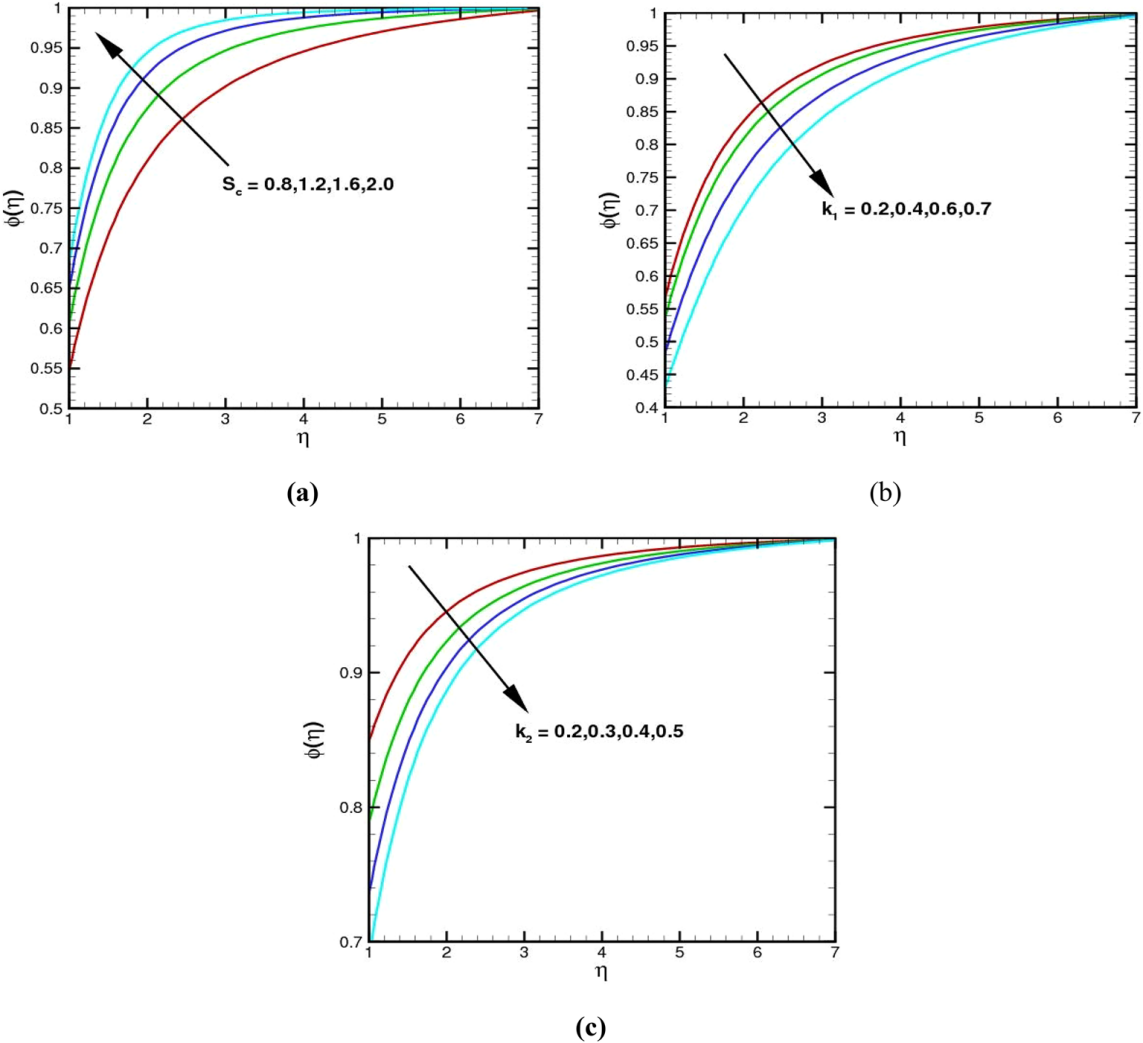
(**a**–**c**) The $\phi \left(\eta \right)$ via ${S}_{c}$, ${k}_{1}$ and ${k}_{2}$, respectively.

The impacts of the Schmidt number ${S}_{c}$, homogeneous parameter ${k}_{1}$, and heterogeneous parameter ${k}_{2}$ on concentration fields are reported in Fig. 7a–c. The ${S}_{c}$ is a ratio of the momentum diffusivity of a fluid to its mass diffusivity. As the value of the ${S}_{c}$ upsurges, the concentration of the fluid upturns in Fig. 7a. An increase in values ${S}_{c}$ indicates that the momentum diffusivity of the fluid increases, which leads to an increase in the concentration profile. In Fig. 7b, it is noted that the $\phi \left(\eta \right)$ decrease for greater values of homogeneous number ${k}_{1}$. Physically, for greater values of ${k}_{1}$, the constructive reaction in the fluid decreases the mass transfer. Figure 7c further demonstrates the influence of ${k}_{2}$ on the concentration profile. Here, a reduction in species concentration is observed for a higher estimation of ${k}_{2}$. It is also noted that the physical behaviour of the homogeneous and heterogeneous responses is consistent.

The validity of this investigation is demonstrated in Table 1, where the obtained results are the best match with the findings of previous research. In Table 2, the numerical results of the surface thermal gradient are determined for various physical parameter values. It is noted that the upsurge in the magnetic field number causes a reduction in the rate of heat transfer; however, the Reynolds parameter, Biot number, and Prandtl number exhibit the opposite trend.

**Table 1:** Axial $f^{{\prime}{\prime}}(1)$ and swirl $g^{\prime}(1)$ velocity gradients comparison values of for various $Re$ when ${\beta }_{1} = M = 0$.

$Re$	$f^{{\prime}{\prime}}(1)$		$g^{\prime}(1)$	
	Ref.^14^	Present	Ref.^14^	Present
0.1	− 0.48960	− 0.4896012	− 0.51023	− 0.5102309
0.2	− 0.61425	− 0.6142532	− 0.52750	− 0.5275013
0.5	− 0.88702	− 0.8870254	− 0.58571	− 0.5857130
1.0	− 1.17963	− 1.1796310	− 0.68797	− 0.6879765
2.0	− 1.59399	− 1.5939980	− 0.87319	− 0.8731950
5.0	− 2.41748	− 2.4174843	− 1.28467	− 1.2846796
10	− 3.34445	− 3.3444567	− 1.81019	− 1.8101976

**Table 2:** $Nu$ for various values of emerging parameters such as $\beta =0.01$, ${\beta }_{t}=0$, ${S}_{c}=1.0$, ${k}_{1}=0.1$, ${\mathrm{k}}_{2}=0.5$.

$Pr$	$Re$	$M$	$\gamma$	$Nu$
2.0	0.01	0.01	0.2	0.7363146
2.5				0.7601068
3.0				0.7783884
2.0	3.0			0.7699975
	4.0			0.7925048
	5.0			0.8090123
	2.0	0.1		0.7409156
		0.3		0.7385680
		0.6		0.7352207
		0.5	0.7	0.9324871
			0.9	1.0944860
			1.1	1.2305260

## Conclusions

We analyzed the mass and heat transport in the Maxwell fluid rotating above the stretching and spinning cylinder and magnetic flux. The novelty of the current problem was to investigate homogeneous–heterogeneous reactions and the Cattaneo–Christov theory to predict the solutal and thermal energy transport mechanisms. Convective conditions were also considered at the boundary. The nonlinear physical problem representing the set of systems of ODEs was numerically integrated through the bvp4c scheme in Matlab 9.7 R2019b. The impacts of some governing parameters, namely the Maxwell parameter, magnetic number, Reynolds number, Prandtl parameter, Biot number, chemical reaction parameters and Schmidt number, on the flow, thermal and concentration profiles were graphically presented and discussed. The analysis leads to the following conclusions:

- The velocity was reduced in magnitude with the upsurge of the Maxwell parameter.
- The Reynolds parameter with a higher trend decreased the velocity and temperature profiles and increased the concentration distributions.
- Greater values of the magnetic number reduced the velocity field's radial, axial, and azimuthal components.
- The thermal relaxation time and Prandtl parameters were effective in lowering the fluid temperature.
- Chemical processes that are both homogeneous and heterogeneous have been discovered to have significant effects on lowering the concentration distribution.

The contribution of this work can lead to new lines of inquiry in the area of thin film flow over a rotating cylinder with some alternative stochastic numerical computing^50–55^.

We believe that the recent upshots will deal with noteworthy information for complex issues within computer routines involving rotating Maxwell fluid with improved heat conduction and chemical reactions because of their numerous applications in processes of heat transfer, heat exchanger, biological processes, combustion, etc., and also utilize these results in experimental studies.

## Data Availability

All data generated or analysed during this study are included in this published article.

## List of symbols

$$u, v , w$$: Velocity components (m s^−^^1^)
$$r, \varphi , z$$: Polar coordinates (m)
$${R}_{1}$$: Cylinder radius (m)
**V**: Velocity field
E: Rotating velocity
$$(a, b)$$: Species concentrations
$$Pr$$: Prandtl parameter
${c}_{p}$: Specific heat capacity
$$\beta$$: Maxwell parameter
$${T}_{\infty }$$: Ambient temperature
${D}_{A}$,${D}_{B}$: Diffusion coefficient parameters
$\sigma$: Electric conductivity of the fluid
$\delta$: Diffusion coefficients ratio parameter
${k}_{1}$: Homogeneous reaction parameter
${k}_{2}$: Heterogeneous reaction parameter
${f}^{\prime}\left(\eta \right)g\left(\eta \right),f(\eta )$: Dimensionless axial, tangential and radial velocities
$${\varvec{B}}$$: Magnetic field
$M$: Magnetic field number
$${{\varvec{J}}}_{1}$$: Current density
($${k}_{c}, {k}_{s}$$): Rate constants
**q**: Heat flux
${\delta }_{E}, {\beta }_{t}$: Fluid thermal relaxation time and thermal relaxation parameter
$$Re$$: Reynolds parameter
$\rho$: Density of fluid
$$T$$: Fluid temperature
$${T}_{w}$$: Wall temperature
$$\eta$$: Dimensionless variable
$$Sc$$: Schmidt parameter
$$\nu$$: Kinematic viscosity
$\theta(\eta), \phi(\eta)$: Dimensionless temperature and concentration
${\lambda }_{1}$: Fluid relaxation time
